# Dr. Jenner on the Treatment of Ringworm

**Published:** 1854-07

**Authors:** 


*Dr. Jenner on the treatment of Ringworm by the local application of Sulphurous Acid.*—The subjoined case is one of two which formed the subject of a clinical lecture by Dr. Jenner. The therapeutical use of this agent was originally suggested by Prof. Graham, as a possible remedy for cholera, at a time when that disease was said to have its origin in the presence of an entophyte in the intestinal canal. It was first employed by Dr. Jenner to check fermentation, and to destroy the torulæ cerevisiæ and sarcina goodsirii.

The case exhibiting the beneficial influence of this acid in ringworm, is that of a Jew pedler, æt. 27 years, who had been affected with this disease for nine years, and who had been variously treated in several hospitals without any benefit. He had been badly fed and lodged, and his habits were sordid, but his general health was pretty good. He was admitted into University College Hospital on March 21st, 1853.

The whole of the scalp (proceeds Dr. Jenner,) excepting the margin, was covered with the crusts of tinea favosa. The largest crusts were of a grayish yellow color, of the consistence of dried putty or mortar, and brittle. Their thickness generally was considerable. Where thickest, the surface of the crust was below the level of the cutis; so that it looked, at the first glance, as if the latter had been partially destroyed by ulceration. The surface of these crusts was very irregular; it had a pitted, worm-eaten, or eroded appearance. At the edge of the large, irregularly-shaped crusts, were many small circular crusts, depressed in the center. A hair passed through the center of each of these small crusts. When the crusts were forcibly detached from the scalp by mechanical means, the exposed surface of the cutis was very red and raw.

The head itched much: and, though scratching gave considerable pain, it was evident, from the traces of blood on the surface, that he had been applying his nails to the part.

The odor of the head was very offensive, something like that emitted by mice, only sweeter and more nauseous. Scattered over the trunk and extremities were a very large number of circular favus crusts. There were as many as forty on the back alone. The smallest of these appeared, when seen through a lens, to be constituted thus: in the center was a hair, around and touching that a brownish-yellow crust, and around that again a dusky-red halo; the diameter of the whole not exceeding two-thirds of a line. On the back no crust was more than one-fourth of an inch in diameter; on the leg there was one one-third of an inch in diameter. These crusts were circular, raised about a line above the level of the cutis, hard, dry, and appeared as though made up of concentric rings of pale, grayish-yellow and brown colors alternating. The surface of these crusts was readily detached, and then a cup-shaped cavity was exposed, filled with a brimstone-yellow powder. The base of the crust being removed, the surface of the cutis, from which it had been detached, was raw.

I saw the mycelium, sporule-bearing branches, and sporules of the achorion Schonleii, when portions of the crusts, or of the yellow powder, were placed under the microscope.

*No treatment was adopted for some time after the man's admission. On April 13th, his state was exactly the same as when he entered the hospital. Rags wet with a solution of sulphurous acid, were now ordered to be kept constantly on the scalp; the head to be covered with an oil-silk cap.*

On April 18th, large quantities of the crust had separated from the scalp, and *those that remained attached had entirely lost their yellow hue; they were now of a dirty-brown color.* All itching of the scalp ceased shortly after the application of the sulphurous acid. No sulphurous acid had been applied to the crusts on the trunk and extremities, and they had still the characters they presented on the man's admission into the hospital.

A piece of lint wet with sulphurous acid lotion, was applied to one of the largest crusts on the leg.

On the 22d April, a mere trace of the favus crust remained on the scalp; but the surface of the cutis was red, and there was an inflamed papula near the vertex. Thinking this condition might be partly due to the acid, which was a very strong solution, I ordered its use to be discontinued for twenty-four hours. The crust on the leg to which the sulphurous acid was applied on the 19th, had separated; the exposed surface was red, but not raw. *Two favus crusts which were seated in the vicinity of that to which the acid was applied on the 19th, were observed to be turning brown; subsequently they dropped off spontaneously.* The effect of the sulphurous acid gas on these two patches is of great interest, as illustrating the mode of action of the solution. The crusts on the scalp turned brown shortly after the acid was applied to them, and before they separated from the cutis.

On the 29th April the lotion was discontinued, and zinc ointment applied to the scalp.

On May 2d the head was free from crusts, but the scalp was still red, and several inflamed papula were seated on it.

On May 9th the skin of the scalp was here and there more natural in hue, and one or two papula had suppurated; the pus was healthy in appearance, and there was no trace of the parasitic plant to be detected by the microscope.

On the 18th, the head continued free from favus; the scalp was much less red; the hair was growing. As the crusts on the trunk and extremities were still in the same state as on the patient's admission into the hospital, he was immersed, about nine in the evening, for half an hour, in a full-sized tepid bath, containing sixteen ounces of saturated solution of sulphurous acid: no friction was employed. During the night all the crusts save three fell from the surface.

On the 20th he was again immersed in the acid bath, and the next day no trace of a crust was to be found on the trunk or extremities. My notes say—"No fresh crusts on head; a small pustule occasionally appears, and dries up in two or three days, and then disappears entirely; the skin of the head generally is much paler and more healthy in aspect."

31st.—The scalp was still paler than at the previous report. There were only two small pustules on the scalp. By the microscope, no trace of the parasite could be detected. The skin generally appeared healthy; and, on June 2d, Jacob left the hospital, at his own desire, to return to Holland.

I cannot conclude without expressing my confident belief that a very great advance was made in pathology when the vegetable nature of the diseases I have referred to as well as of some others, was demonstrated; and my equally confident belief, that the foundation for a very great advance in therapeutics was laid when Professor Graham introduced to notice the power of sulphurous acid to destroy vegetable life, and explained how it could be given internally without injury to the patient.

Note.—The solution of sulphurous acid I have used is made by passing a stream of the gas through water till the latter is saturated. Of this saturated solution, two ounces may be added to six ounces of water, to make the lotion.

The saturated solution of sulphuric acid I have employed, was prepared in the Birbeck Laboratory of University College.

